# Comparative genomic analysis of C4 photosynthetic pathway evolution in grasses

**DOI:** 10.1186/gb-2009-10-6-r68

**Published:** 2009-06-23

**Authors:** Xiyin Wang, Udo Gowik, Haibao Tang, John E Bowers, Peter Westhoff, Andrew H Paterson

**Affiliations:** 1 Plant Genome Mapping Laboratory, University of Georgia, Athens, GA 30602, USA; 2 College of Sciences, Hebei Polytechnic University, Tangshan, Hebei 063000, China; 3 Institut fur Entwicklungs- und Molekularbiologie der Pflanzen, Heinrich-Heine-Universitat 1, Universitatsstrasse, D-40225 Dusseldorf, Germany; 4 Department of Plant Biology, University of Georgia, Athens, GA 30602, USA

## Abstract

**Background:**

Sorghum is the first C4 plant and the second grass with a full genome sequence available. This makes it possible to perform a whole-genome-level exploration of C4 pathway evolution by comparing key photosynthetic enzyme genes in sorghum, maize (C4) and rice (C3), and to investigate a long-standing hypothesis that a reservoir of duplicated genes is a prerequisite for the evolution of C4 photosynthesis from a C3 progenitor.

**Results:**

We show that both whole-genome and individual gene duplication have contributed to the evolution of C4 photosynthesis. The C4 gene isoforms show differential duplicability, with some C4 genes being recruited from whole genome duplication duplicates by multiple modes of functional innovation. The sorghum and maize carbonic anhydrase genes display a novel mode of new gene formation, with recursive tandem duplication and gene fusion accompanied by adaptive evolution to produce C4 genes with one to three functional units. Other C4 enzymes in sorghum and maize also show evidence of adaptive evolution, though differing in level and mode. Intriguingly, a phosphoenolpyruvate carboxylase gene in the C3 plant rice has also been evolving rapidly and shows evidence of adaptive evolution, although lacking key mutations that are characteristic of C4 metabolism. We also found evidence that both gene redundancy and alternative splicing may have sheltered the evolution of new function.

**Conclusions:**

Gene duplication followed by functional innovation is common to evolution of most but not all C4 genes. The apparently long time-lag between the availability of duplicates for recruitment into C4 and the appearance of C4 grasses, together with the heterogeneity of origins of C4 genes, suggests that there may have been a long transition process before the establishment of C4 photosynthesis.

## Abstract 2

Comparison of the sorghum, maize and rice genomes shows that gene duplication and functional innovation is common to evolution of most but not all genes in the C4 photosynthetic pathway

## Background

Many of the most productive crops in agriculture use the C4 photosynthetic pathway. Despite their multiple origins, they are all characterized by high rates of photosynthesis and efficient use of water and nitrogen. As a morphological and biochemical innovation [1], the C4 photosynthetic pathway is proposed to have been an adaptation to hot, dry environments or CO_2 _deficiency [2-5]. The C4 pathway independently appeared at least 50 times during angiosperm evolution [6,7]. Multiple origins of the C4 pathway within some angiosperm families [8,9] imply that its evolution may not be complex, perhaps suggesting that there may have been genetic pre-deposition in some C3 plants to C4 evolution [6].

**Figure 1:**
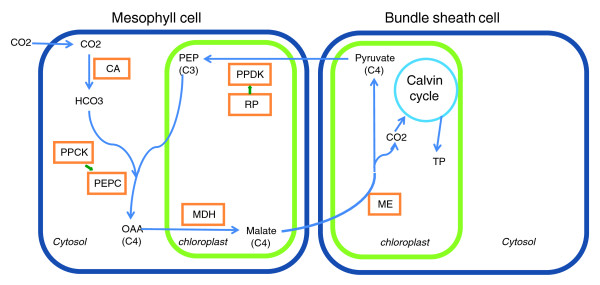
**The NADP-ME type of C4 pathway in sorghum and maize**. CA, carboxylating anhydrase; MDH, malate dehydrogenase; ME, malic enzyme; OAA, oxaloacetate; PEPC, phosphoenolpyruvate carboxylase; PPCK, PEPC kinase; PPDK, pyruvate orthophosphate dikinase; PPDK-RP, PPDK regulatory protein; TP, transit peptide.

The high photosynthetic capacity of C4 plants is due to their unique mode of CO_2 _assimilation, featuring strict compartmentation of photosynthetic enzymes into two distinct cell types, mesophyll and bundle-sheath (illustrated in Figure 1 for the NADP-malic enzyme (NADP-ME) type of C4 pathway). First, CO_2 _assimilation is carried out in mesophyll cells. The primary carboxylating enzyme, phosphoenolpyruvate carboxylase (PEPC), together with carbonic anhydrase (CA), which is crucial to facilitating rapid equilibrium between CO_2 _and , is responsible for the hydration and fixation of CO_2 _to produce a C4 acid, oxaloacetate. In NADP-ME-type C4 species, oxaloacetate is then converted to another C4 acid, malate, catalyzed by malate dehydrogenase (MDH). Malate then diffuses into chloroplasts in the proximal bundle-sheath cells, where CO_2 _is released to yield pyruvate by the decarboxylating NADP-ME. The released CO_2 _concentrates around the secondary carboxylase, Rubisco, and is reassimilated by it through the Calvin cycle. Pyruvate is transferred back into mesophyll cells and catalyzed by pyruvate orthophosphate dikinase (PPDK) to regenerate the primary CO_2 _acceptor, phosphoenolpyruvate. Phosphorylation of a conserved serine residue close to the amino-terminal end of the PEPC polypeptide is essential to its activity by reducing sensitivity to the feedback inhibitor malate and a catalyst named PEPC kinase (PPCK). C4 photosynthesis results in more efficient carbon assimilation at high temperatures because its combination of morphological and biochemical features reduce photorespiration, a loss of CO_2 _that occurs during C3 photosynthesis at high temperatures [10]. PPDK regulatory protein (PPDK-RP), a bifunctional serine/threonine kinase-phosphatase, catalyzes both the ADP-dependent inactivation and the Pi-dependent activation of PPDK [11].

The evolution of a novel biochemical pathway is based on the creation of new genes, or functional changes in existing genes. Gene duplication has been recognized as one of the principal mechanisms of the evolution of new genes. Genes encoding enzymes of the C4 cycle often belong to gene families having multiple copies. For example, in maize and sorghum, a single C4 PEPC gene and other non-C4 isoforms were discovered [12], whereas in *Flaveria trinervia*, a C4 eudicot, multiple copies of C4 PEPC genes were found [13]. These findings led to the proposition that gene duplication, followed by functional innovation, was the genetic foundation for photosynthetic pathway transformation [14].

All plant genomes, including grass genomes, have been enriched with duplicated genes derived from tandem duplications, single-gene duplications, and large-scale or whole-genome duplications [15-18]. A whole-genome duplication (WGD) occurred in a grass ancestor approximately 70 million years ago (mya), before the divergence of the panicoid, oryzoid, pooid, and other major cereal lineages [19,20]. A preliminary analysis of sorghum genome data suggested that duplicated genes from various sources have expanded the sizes of some families of C4 genes and their non-C4 isoforms [21]. However, different duplicated gene pairs often have divergent fates [22]. While most duplicated genes are lost, gene retention in some functional groups produces large gene families in plants [15,19,20]. Together with other lines of evidence, these have led to the interesting proposition of differential gene duplicability [23,24], or duplication-resistance [25], due to possible gene dosage imbalance, which can be deleterious [26]. Even when duplicated genes survive, there is rarely strong evidence supporting possible functional innovation [27].

Most C4 plants are grasses, and it has been inferred that C4 photosynthesis first arose in grasses during the Oligocene epoch (24 to 35 mya) [28,29]. Sorghum and maize, thought to have diverged from a common ancestor approximately 12 to 15 mya [21], are both in the Andropogoneae tribe, which is entirely composed of C4 plants [8]. Sorghum, a NADP-ME-type C4 plant grown for food, feed, fiber and fuel, is the second grass and the first C4 plant with its full genome sequence available [21]. The first grass genome sequenced was rice, a C3 plant. The availability of two grass genome sequences using different types of photosynthesis provides a valuable opportunity to explore C4 pathway evolution. In the present research, by using a comparative genomic approach and phylogenetic analysis, we compared C4 genes and their non-C4 isoforms in sorghum, maize and rice. The aims of this study are to investigate: the role of gene duplication in the evolution of C4 enzyme genes; the role of adaptive evolution in C4 pathway formation; the long-standing hypothesis that a reservoir of duplicated genes has been a prerequisite of C4 pathway evolution [14]; and whether codon usage bias has contributed to C4 gene evolution, as previously suggested [30]. Our results will help to clarify the evolution of the C4 pathway and may benefit efforts to transform C3 plants, such as rice, to C4 photosynthesis [31].

## Results

### PEPC enzyme genes

Grass PEPC enzyme genes form a small gene family. There are five plant-type and one bacteria-type PEPC (Sb03g008410 and Os01g0110700) [32] gene isoforms in sorghum and rice, respectively, excepting two likely pseudogenized rice isoforms (Os01g0208800, Os09g0315700) having only 217 and 70 codons. There is one sorghum C4 PEPC [33,34], Sb10g021330 (Table S1 in Additional data file 1). Previous characterization indicated that its transcripts are more than 20 times more abundant in mesophyll than in bundle-sheath cells [35] (Table S2 in Additional data file 1).

By analysis of gene colinearity, we investigated how genome duplication has affected the PEPC gene families in rice and sorghum. The PEPC gene in rice that is most similar to the sorghum C4 PEPC is Os01g0208700, sharing 73% amino acid identity. This similarity raised the possibility that the two genes are orthologous. Although the two genes under consideration are not in colinear locations, single-gene translocation is not rare in grasses [36]. The outparalogs, homologs produced by WGD in the common ancestor of sorghum and rice, of the sorghum C4 PEPC gene are located at the expected homoeologous locations in both sorghum and rice (Sb04g008720 and Os02g0244700). The rice gene Os01g0208700 and the C4 genes are grouped together, and outparalogs (Os02g0244700 and Sb04g008720) of the sorghum C4 gene form a sister group on the phylogenetic tree. The pattern can be explained if Os01g0208700 were orthologous to the sorghum C4 PEPC gene, implied by their high sequence similarity and shared high GC content (detailed below). In our view, the most parsimonious explanation of these data is that the oryzoid (rice) ortholog was translocated after the sorghum-rice (panicoid-oryzoid) divergence, then the panicoid (sorghum) ortholog was recruited into the C4 pathway. We cannot falsify a model invoking independent loss of alternative homeologs in sorghum (panicoids) and rice (oryzoids), respectively, although this model seems improbable in that such loss of alternative homoeologs has only occurred for approximately 1.8 to 3% of genome-wide gene duplicates in these taxa [21]. The other rice and sorghum PEPC genes form four orthologous pairs. Whether the genes from different orthologous groups are outparalogs could not be supported by colinearity inference associated with the pan-cereal genome duplication.

Grass PEPC genes show high GC content, like many other grass genes, apparently as a result of changes after the monocot-dicot split but before the radiation of the grasses [37]. The evolution of C4 PEPC genes in sorghum and maize was previously proposed to have been accompanied by GC elevation, resulting in codon usage bias [38]. We found that C4 PEPC genes do have higher GC content than other sorghum and maize PEPC genes, especially at the third codon sites (GC3). The sorghum and maize C4 PEPC genes have a GC3 content of approximately 84%, significantly higher than other genes in both species (Table S3 in Additional data file 1). The suspected rice ortholog Os01g0208700 has even higher GC3 content, approximately 92%. In contrast, the GC3 content of all *Arabidopsis *PEPC genes is <43%. This shows that the higher GC content in the C4 PEPC genes may not be related to the evolution of C4 function, as discussed below.

**Figure 2:**
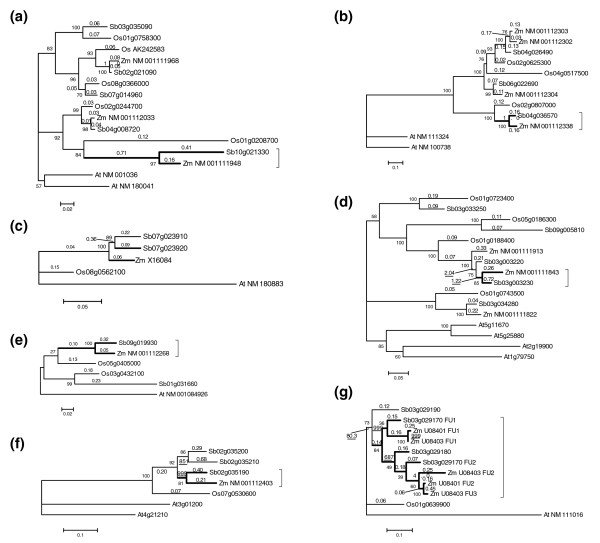
**Phylogeny of C4 enzyme genes and their isoforms in sorghum, rice, maize and *Arabidopsis***. Thick branches show C4 enzyme genes. Bootstrap percentage values are shown as integers; Ka/Ks ratios are shown as numbers with fractions, or underlined when >1. In the gene IDs, Sb indicates *Sorghum bicolor*, Os indicates *Oryza sativa*, Zm indicates *Zea mays*, and At indicates *Arabidopsis thaliana*. **(a) **PEPC; **(b) **PPCK; **(c) **NADP-MDH; **(d) **NADP-ME; **(e) **PPDK; **(f) **PPDK-RP; **(g) **CA.

C4 PEPC genes show evidence of adaptive evolution. To characterize the evolution of C4 PEPC genes, we aligned the sequences and constructed gene trees without involving the possible pseudogenized rice gene (Additional data file 2). We found the genes to be in two groups, with one containing plant-type and the other bacteria-type PEPC genes. Careful inspection suggested problems with the tree, for orthologous genes were not grouped together as expected. After removing the bacteria-type genes and rooting the subtree containing the C4 genes with *Arabidopsis *PEPC genes, we obtained a tree in which orthologs are grouped together as expected (Figure 2a). The sorghum and maize C4 genes are on a remarkably long branch, suggesting that they are rapidly evolving compared to the other genes, and implying possible adaptive selection during the evolution of the C4 pathway, consistent with a previous proposal [39].

Maximum likelihood analysis supports possible adaptive evolution of C4 PEPC genes. First, characterization of nonsynonymous nucleotide substitution rates (Ka) supports rapid evolution of the C4 genes and their rice ortholog. Under a free-parameter model, Ka values are >0.048 on branches leading to C4 genes and their rice ortholog after the rice-sorghum split, as compared to ≤0.02 on branches leading to the non-C4 isoforms. Second, the C4 genes may have been positively selected. The Ka/Ks ratio is nearly tenfold higher (0.71) on the branch leading to the last common ancestor of the sorghum and maize C4 genes than on other branches after the rice-sorghum split (≤0.08). Though the ratio is <1, we propose that the striking difference in Ka/Ks between C4 and non-C4 genes may be evidence of positive selection in the C4 genes for the following reasons: the criterion Ka/Ks > 1 has been proposed to be unduly stringent to infer positive selection [40]; the maximum likelihood analysis is conservative, as reported previously [27]; and the similar slow evolutionary changes in all non-C4 genes in sorghum, maize and rice (Figure 1a) imply elevated rates in the C4 genes, rather than purifying selection in the non-C4 genes.

C4 PEPC genes show elevated and aggregated amino acid substitutions especially in function-specific regions, providing further evidence of adaptive evolution. Comparison to their outparalogs and their nearest outgroup sequence suggests that C4 PEPC genes have accumulated approximately 100 putative substitutions over their full length (Table 1), far more than non-C4 PEPC genes. The substitutions are referred to as putative since we cannot rule out the possibility of parallel and reverse mutations. However, the extremely significant difference strongly supports divergent evolution of C4 and non-C4 PEPC genes. The amino acid substitutions are not uniformly distributed along the lengths of the C4 genes (Table S4 in Additional data file 1), but concentrated in the carboxy-terminal half, including the critical mutation S780 (the serine at position 780 of the maize C4 PEPC protein that is essential to relieving feedback inhibition by malate [41]). This is consistent with previous findings [42].

**Table 1:** Aggregated amino acid substitution analysis results

Gene 1	Gene 2	Outgroup	Alignment length	Alignment length without gaps	Average identity	Overall substitution number in gene 1	Overall substitution number in gene 2	*P*-value
**PEPC**								
Sb10g021330	Os02g0244700	Os01g0758300	972	958	0.76	110	26	5.89E-13
Zm_NM_00111968	Os02g0244700	Os01g0758300	971	968	0.78	92	33	1.31E-07
Sb10g021330	Os02g0244700	Sb03g035090	972	958	0.76	117	28	1.46E-13
Zm_NM_00111968	Os02g0244700	Sb03g035090	971	968	0.77	104	34	2.54E-09
								
**PPCK**								
Sb04g036570	Os02g0807000	Sb06g022690	309	284	0.65	15	14	8.53E-01
Zm_NM_001112338	Os02g0807000	Sb06g022690	309	281	0.63	18	11	1.94E-01
								
**CA**								
U08403_FU3	Os01g0639900	Sb03g029190.1	272	201	0.75	19	18	8.69E-01
U08403_FU2	Os01g0639900	Sb03g029190.1	273	200	0.73	20	18	7.46E-01
U08403_FU1	Os01g0639900	Sb03g029190.1	273	202	0.79	13	18	3.69E-01
U08401_FU2	Os01g0639900	Sb03g029190.1	272	201	0.75	18	18	1.00E+00
U08401_FU1	Os01g0639900	Sb03g029190.1	273	202	0.78	14	18	4.80E-01
Sb03g029170_FU2	Os01g0639900	Sb03g029190.1	272	201	0.78	14	16	7.15E-01
Sb03g029170_FU1	Os01g0639900	Sb03g029190.1	273	201	0.80	11	20	1.06E-01
Sb03g029180	Os01g0639900	Sb03g029190.1	274	202	0.80	11	19	1.44E-01
U08403_FU3	Os01g0639900	At_NM_111016	293	201	0.50	14	13	8.47E-01
U08403_FU2	Os01g0639900	At_NM_111016	293	200	0.49	16	14	7.15E-01
U08403_FU1	Os01g0639900	At_NM_111016	293	202	0.50	10	15	3.17E-01
U08401_FU2	Os01g0639900	At_NM_111016	293	201	0.50	12	13	8.41E-01
U08401_FU1	Os01g0639900	At_NM_111016	293	202	0.50	11	15	4.33E-01
Sb03g029170_FU2	Os01g0639900	At_NM_111016	293	201	0.50	10	10	1.00E+00
Sb03g029170_FU1	Os01g0639900	At_NM_111016	293	201	0.50	9	14	2.97E-01
Sb03g029180	Os01g0639900	At_NM_111016	293	202	0.50	8	11	4.91E-01
								
**PPDK**								
Sb09g019930	Os05g0405000	Os03g0432100	949	946	0.83	42	28	9.43E-02
Zm_NM_001112268	Os05g0405000	Os03g0432100	950	944	0.83	44	28	5.93E-02
Sb09g019930	Os05g0405000	Sb01g031660	958	946	0.76	37	15	2.28E-03
Zm_NM_001112268	Os05g0405000	Sb01g031660	961	942	0.78	32	18	4.77E-02
NADP-MDH								
Sb07g023920	Os08g0562100	At_NM_180883	443	427	0.77	22	19	6.39E-01
Sb07g023910	Os08g0562100	At_NM_180883	443	432	0.75	25	16	1.60E-01
ZM_X16084	Os08g0562100	At_NM_180883	443	430	0.75	25	13	5.16E-02
								
**NADP-ME**								
Sb03g003230	Os01g0188400	Os05g0186300	642	633	0.80	46	16	1.39E-04
Sb03g003230	Os01g0188400	Sb09g005810	642	633	0.80	41	20	7.17E-03
Sb03g003220	Os01g0188400	Os05g0186300	650	635	0.84	23	15	1.94E-01
ZM_NM_001111843	Os01g0188400	Os05g0186300	641	634	0.80	47	16	9.40E-05
ZM_NM_001111913	Os01g0188400	Os05g0186300	668	633	0.84	26	15	8.58E-02
								
**PPDK-RP**								
Sb02g035190	Os07g0530600	At4g21210	474	426	0.58	37	17	6.00E-03
Zm_NM_001112403	Os07g0530600	At4g21210	474	423	0.57	33	23	1.80E-01
Sb02g035190	Sb02g035200	Os07g0530600	476	408	0.69	19	22	6.40E-01
Sb02g035190	Sb02g035210	Os07g0530600	483	384	0.69	21	22	8.70E-01
Zm_NM_001112403	Sb02g035200	Os07g0530600	472	416	0.67	25	22	6.60E-01
Zm_NM_001112403	Sb02g035210	Os07g0530600	482	389	0.68	25	25	1.00E+00

Surprisingly, Os01g0208700 has also accumulated significantly more mutations than expected, and has a relatively larger selection pressure than other non-C4 PEPC genes, implying that it may also be under adaptive selection (Table 1; Table S4 in Additional data file 1), as further discussed below.

### PPCK enzyme genes

PPCK gene families have been enriched by duplication events, including the pan-cereal WGD and tandem duplication. We identified three PPCK gene isoforms in both sorghum and rice, respectively (Table S1 in Additional data file 1), which are in one-to-one correspondence in expected colinear locations between the two species (Figure 2b). These rice and sorghum isoforms correspond to four maize isoforms (ZmPPCK1 to ZmPPCK4; Figure 2b), with ZmPPCK2 and ZmPPCK3 likely produced in maize after its divergence from a lineage shared with sorghum. The sorghum C4 PPCK is encoded by Sb04g036570, and its maize ortholog is ZmPPCK1. Their C4 nature is supported by evidence that their expression is light-induced and their transcripts are more abundant in mesophyll than bundle-sheath cells [30]. In contrast, the expression of sorghum and maize non-C4 isoforms is not light- but cycloheximide-affected [30]. The outparalogs of the sorghum C4 gene and its rice ortholog were likely lost before the two species split, whereas the other four isoforms are outparalogs.

Maximum likelihood analysis and inference of aggregated amino acid substitutions found no evidence of adaptive selection during C4 PPCK gene evolution (Table S4 in Additional data file 1).

Consistent with a previous report [30], all studied grass PPCK genes have extremely high GC content, with a GC3 content from 88 to 97% (Table S3 in Additional data file 1). The grass C4 and non-C4 PPCK genes have similar GC content.

### NADP-MDH enzyme genes

There are two NADP-MDH enzyme genes in sorghum (Table S1 in Additional data file 1), the non-C4 gene Sb07g023910 and the C4 gene Sb07g023920, tandemly located as previously reported [43]. They have only one homolog in both rice and maize [44], with the rice homolog (Os08g0562100) at the expected colinear location. This suggests that the NADP-MDH WGD outparalog was lost before the sorghum-rice split. Each of the sorghum tandem genes has an ortholog in *Vetiveria *and *Saccharum*, respectively [44], suggesting that the tandem duplication occurred before the divergence of sorghum and *Vetiveria*, but after the sorghum-maize split, an inference further supported by gene tree analysis in that they are more similar to one another than to the single maize homolog (Figure 2c).

The C4 NADP-MDH gene shows an interesting mode of adaptive evolution. Though the C4 NADP-MDH genes have accumulated more mutations than non-C4 genes (Table S4 in Additional data file 1), neither maximum likelihood analysis nor the inference of aggregated amino acid substitution suggest adaptive selection. However, the sorghum C3 and C4 genes were likely to have been produced by an ancestral C4 gene through duplication. One of the duplicates may have lost its C4 function as it is not light-induced and only constitutively expressed [43].

The NADP-MDH genes are chloroplastic. A chloroplast transit peptide (cTP) having approximately 40 amino acids is identified in all the genes from grasses and *Arabidopsis *(Additional data file 3). This indicates that the cTP was present in the common ancestor of angiosperms. Non-chloroplastic NADP-MDH genes identified in the sorghum genome share less than 40% protein sequence similarity with the chloroplastic ones.

All of the grass NADP-MDH enzyme genes studied have elevated GC content compared to the *Arabidopsis *ortholog, especially regarding GC3 (50% versus 40%; Table S3 in Additional data file 1). The grass C4 genes have slightly higher GC content than the non-C4 genes.

### NADP-ME enzyme genes

The NADP-ME gene family has been gradually expanding due to tandem duplication and the pan-cereal WGD. We identified five and four NADP-ME enzyme genes in sorghum and rice, respectively (Table S1 in Additional data file 1). The sorghum C4 gene is Sb03g003230, whose transcript is abundant in bundle-sheath but not mesophyll cells [35] (Table S2 in Additional data file 1). The C4 gene has a tandem duplicate that may have been produced before the sorghum-maize split based on gene similarity and tree topology (Figure 2d). The tandem genes share the same rice ortholog (Os01g0188400) at the expected colinear location, and their WGD duplicates can be found at the expected colinear location in both species. The other sorghum and rice NADP-ME genes form two orthologous pairs, having also remained at the colinear locations predicted based on the pan-cereal duplication.

Maximum likelihood analysis indicates that the sorghum and maize C4 NADP-ME genes are under positive selection. The branches leading to their two closest ancestral nodes have a Ka/Ks ratio > 1 (*P*-value = 8 × 10^-10^). Moreover, the C4 genes have a significant abundance of amino acid substitutions (Table 1; Table S4 in Additional data file 1). The most affected regions in sorghum and maize overlap with one another, from residue 141 to residue 230 in sorghum, and from residue 69 to residue 181 in maize.

The grass NADP-ME genes have higher GC content than their *Arabidopsis *homologs (Table S3 in Additional data file 1). The highest GC content (GC3 > 82%) is found not in the C4 genes but in their outparalogs, Sb09g005810 and Os05g0186300.

The C4 genes, their tandem paralogs in sorghum and maize, and their rice ortholog all share an approximately 39 amino acid cTP that is absent from their WGD paralogs in grasses, or homologs in *Arabidopsis*. This seems to suggest that the cTP was acquired by one member of a duplicated gene pair after the pan-grass WGD but before the sorghum-rice divergence.

### PPDK enzyme genes

Sorghum and rice both have two PPDK enzyme genes (Table S1 in Additional data file 1). The sorghum C4 PPDK gene (Sb09g019930) is identified based on its approximately 90% amino acid identity with the maize C4 gene. Its transcript is abundant in mesophyll rather than bundle-sheath cells [35] (Table S2 in Additional data file 1). Its rice ortholog (Os05g0405000) can be inferred based on both gene trees (Figure 2e) and gene colinearity. The other rice and sorghum isoforms are orthologous to one another. Whether the four isoforms are outparalogs produced by the WGD could not be determined by gene colinearity inference due to possible gene translocations. However, synonymous nucleotide substitution rates and gene tree topologies support that the rice and sorghum paralogs were produced before the two species diverged, and approximately at the time of the pan-cereal WGD.

There are two PPDK genes in maize [10]. One of them encodes both a C4 transcript and a cytosolic transcript, controlled by distinct upstream regulatory elements [45]. The C4 copy has an extra exon encoding a cTP at a site upstream of the cytosolic gene [46]. We found that the sorghum C4 PPDK gene is highly similar to its maize counterpart along their respective full lengths, indicating their origin in a common maize-sorghum ancestor. The other maize PPDK gene has only a partial DNA sequence and, therefore, has been avoided in the present evolutionary analysis. A similarity search against the maize bacterial artificial chromosome (BAC) sequences indicates that it is on a different chromosome (chromosome 8) from the C4 gene (chromosome 6). The maize counterpart of the other sorghum PPDK isoform has not yet been identified in sequenced BACs.

The C4 PPDK genes may have experienced adaptive evolution. While maximum likelihood analysis did not find evidence of adaptive evolution of C4 PPDK genes (Figure 2e), the C4 genes have accumulated significantly or nearly significantly more amino acid substitutions than their rice orthologs, particularly in the region from approximately residue 207 to approximately residue 620 (Table 1; Table S4 in Additional data file 1).

All grass PPDK genes have higher GC content than their *Arabidopsis *homologs (Table S3 in Additional data file 1), with the C4 genes themselves being highest in GC content (GC3 content approximately 61 to 70%).

All of the characterized PPDK isoform sequences from grasses and Arabidopsis share an approximately 20 amino acid cTP (Additional data file 3), suggesting its origin before the monocot-dicot split.

### PPDK-RP enzyme genes

Tandem duplication contributed to the expansion of PPDK-RP genes. Using the maize PPDK-RP gene sequence as a query, we determined its possible sorghum ortholog, Sb02g035190, which has two tandem paralogs. Their rice ortholog, Os07g0530600, was identified in the anticipated colinear region. However, we failed to find their WGD outparalogs in both sorghum and rice, suggesting possible gene loss in their common ancestor.

Gene trees indicate that the tandem duplication events may have occurred before the sorghum-maize divergence, but after the sorghum-rice divergence (Figure 2f). Maximum likelihood analysis suggests that both lineages leading to the maize PPDK-RP gene and its sorghum ortholog, and other isoforms, have been under significant positive selection (Ka/Ks >> 1, *P*-value = 2.5 × 10^-8^), implying possible functional changes in both lineages. Compared to their rice ortholog, sorghum and maize PPDK-RP genes have accumulated significantly more amino acid substitutions (Table 1; Table S4 in Additional data file 1), providing supporting evidence for functional innovation.

Both the C4 and non-C4 PPDK-RP genes in sorghum have similar GC content (GC3 content approximately 57 to 60%), while the maize PPDK-RP gene has higher GC content (GC3 content approximately 67%), especially in the third codon sites (Table S3 in Additional data file 1). All these grass PPDK-RP genes show higher GC content than their *Arabidopsis *homologs.

### CA enzyme genes

**Figure 3:**
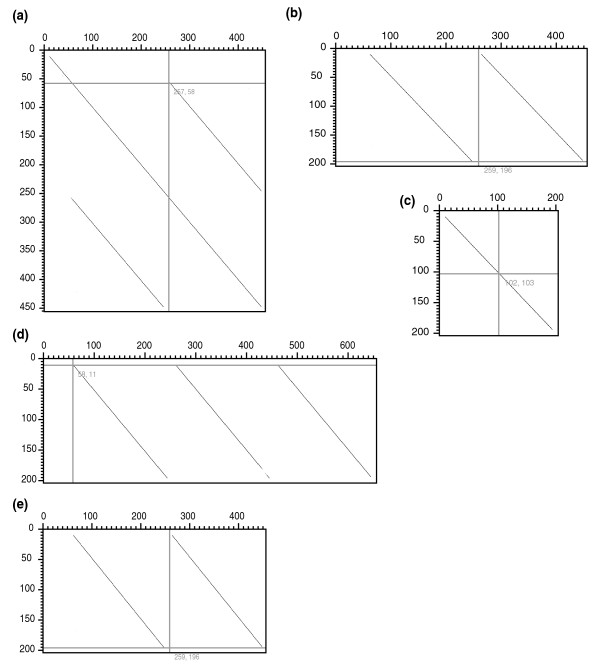
**Dotplots between sorghum and maize CA enzyme protein sequences**. **(a) **Self-comparison of protein sequence of Sb03g029170. **(b) **Sb03g029170 (horizontal) and Sb03g029180 (vertical); **(c) **Sb03g029190 (horizontal) and Sb03g029180 (vertical); **(d) **maize U08403 (horizontal) and Sb03g029180 (vertical); **(e) **maize U08401 (horizontal) and Sb03g029180 (vertical).

Tandem duplication has profoundly affected the evolution of CA genes. There are two types of CA enzymes, the alpha and beta types in sorghum [21], and C4 CA genes are the beta type [47]. Here, we focus on beta-type CA genes. Our analysis indicates that there are four beta-type CA enzyme gene isoforms in sorghum, forming a tandem gene cluster with the same transcriptional orientation, on chromosome 3 (Figure 3a; Table S1 in Additional data file 1). Among them are two possible C4 genes (Sb03g029170 and Sb03g029180), which were shown by previous analysis of transcript abundance to be highly expressed in mesophyll but not bundle-sheath cells (Table S2 in Additional data file 1). The other two genes include one non-C4 gene (Sb03g029190) and one probable pseudogene (Sb03g029200) with only truncated coding sequence, a large DNA insertion in its second exon, and accumulated point mutations. These tandem genes have a common rice ortholog (Os01g0639900) at the expected colinear location, indicating that gene family expansion has occurred in sorghum (and maize; see below) since divergence from rice. The WGD outparalogs were not identified in either genome, implying possible gene loss after the WGD and before the rice-sorghum split.

**Figure 4:**
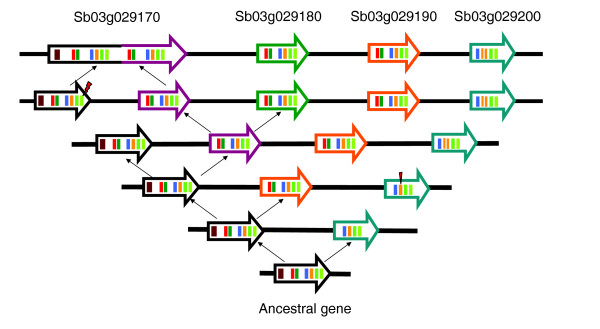
**Tandem duplication and fusion of CA genes in sorghum**. Postulated evolution of sorghum CA genes through four tandem duplication events and a gene fusion event is displayed. We show distribution and structures of CA genes, and their peptide-encoding exons, on sorghum chromosome 3. Genes are shown as the large arrows with differently colored outlines and exons are shown as colored blocks contained in the arrows. Homologous exons are in the same color. A chloroplast transit peptide is in dark red. A tandem duplication event is shown by two small black arrows pointing in divergent directions, and a gene fusion event is shown by two small black arrows pointing in convergent directions. A new gene produced by tandem duplication is shown with an arrow in a new color not used by the ancestral genes. A gene produced by fusion of two neighboring genes is shown as a bipartite structure, each part with the color of one of the fused genes. A stop codon mutation is shown by a lightning-bolt symbol, and an exon-splitting event by a narrow triangle.

The two sorghum C4 CA genes differ in cDNA length [35]. We found that the larger C4 CA gene may have evolved by fusing two neighboring CA genes produced by tandem duplication. In spite of possible alternative splicing programs, Sb03g029170 has a gene length of approximately 10.4 kbp and includes 13 exons, as compared to 4.5 kbp in length and 6 exons for Sb03g029180. Pairwise dotplots between Sb03g029170 and Sb03g029180 show the former has an internal repeat structure absent from the latter (Figure 3ab; Additional data file 4). The duplication involves the last six of seven exons and intervening introns 1 to 6 of the ancestral gene (Figure 4a). Comparatively, the other sorghum genes have only exons 2 to 7, assumed to be a functional unit, both lacking the first exon in Sb03g029170, which encodes a cTP. This implies that several duplication events have recursively produced extra copies of the functional unit. Some functional units act as independent genes, while the other fused with the complete one to form an expanded gene including two functional units. We found that this fusion involved mutation of the stop codon in the leading gene. Each functional unit starts with an ATG codon, which we infer may increase the possibility of alternative splicing. This inference is supported by the finding that Sb03g029170 may have two distinct transcripts, identified by cDNA HHU69 and HHU22, respectively (Table S2 in Additional data file 1). The two transcripts have distinct lengths, 2,100 and 1,200 bp, respectively, with the expression of the longer one being light-inducible and C4-related but the shorter one not [35]. The non-C4 gene, Sb03g029190, has a normal structure (Figure 3c) and the pseudogene, Sb03g029200, has a truncated structure.

The tandem duplication and gene fusion are shared by sorghum and maize, and maize furthermore has additional duplication. Interestingly, we found that the maize CA enzyme genes have two and three functional units, respectively (Figure 3de; Additional data file 4), implying further DNA sequence duplication and gene fusion in the maize lineage. Mutation of stop codons was also found in the leading gene sequences. Rice and *Arabidopsis *genes have only one functional unit preceded by a cTP.

To clarify the evolution of CA genes, we performed a phylogenetic analysis of the functional units (Figure 2f). The first functional units from sorghum and maize genes are grouped together, the second and third maize units and that of Sb03g029180 were in another group, and the rice gene and non-C4 sorghum gene Sb03g029190 were outgroups. This suggests the origin of the extra functional units to be after the Panicoideae-Ehrhartoideae divergence but before the sorghum-maize divergence, and continuing in the maize lineage. A possible evolutionary process in sorghum is illustrated in Figure 4b.

A gene tree of functional units suggested that C4 CA genes may have been affected by positive selection. According to the free-parameter model of the maximum likelihood approach, we found that the two functional unit groups revealed above may have experienced positive selection, in that Ka/Ks > 1 (Figure 2f), though this possibility is not significantly supported by statistical tests or by amino acid substitution analysis (Table S4 in Additional data file 1).

Excepting the possibly pseudogenized sorghum CA gene, the grass isoforms have very high GC content (GC3 content 82 to 92%), much higher than that of the *Arabidopsis *orthologs (Table S3 in Additional data file 1). The non-C4 gene, Sb03g029190, rather than any of the C4 genes, has the highest GC content in sorghum.

## Discussion

### Gene duplication and C4 pathway evolution

In the case of the C4 pathway, the evolution of a novel biological pathway required the availability of gene families with multiple members, in which modification of both expression patterns and functional domains led to new adaptive phenotypes. An intuitive idea is that genetic novelty formation is simplified by exploiting available 'construction bricks', and the pathway genes that we are aware of were either 'subverted' from existing functions or were created through modification of existing genes. Three mechanisms of new gene formation have been proposed [48]: duplication of pre-existing genes followed by neofunctionalization; creation of mosaic genes from parts of other genes; and *de novo *invention of genes from DNA sequences.

Duplicated genes have long been suggested to contribute to the evolution of new biological functions. As early as 1932, Haldane suggested that gene duplication events might have contributed new genetic materials because they create initially identical copies of genes, which could be altered later to produce new genes without disadvantage to the organism [49]. Ohno proposed that gene duplication played an essential role in evolution [50], pointed out the importance that WGD might have had on speciation, and hypothesized that at least one WGD event facilitated the evolution of vertebrates [51]. This hypothesis has been supported by evidence from various gene families, and from the whole genome sequences of several metazoans [52,53]. Plant genomes have experienced recurring WGDs [15,54-57], and perhaps all angiosperms are ancient polyploids [54]. These polyploidy events contribute to the creation of important developmental and regulatory genes [58-61], and may have played an important role in the origin and diversification of the angiosperms [62]. About 20 million years before the divergence of the major grass clades [19,20], the ancestral grass genome was affected by a WGD, possibly preceded by still more ancient duplication events [17,63]. It is tempting to link this WGD to the evolutionary success of grasses, now including more than 10,000 species and covering about 20% of the Earth's land surface [64], though such a link has not yet been adequately justified.

Gene duplication has been related to the evolution of the C4 pathway, based on the finding that C4 enzyme genes are usually from families having multiple copies [14]. Consequently, an ability to create and maintain large numbers of duplicated genes has been supposed to be one precondition for certain taxa to develop C4 photosynthesis [6,14]. It was even suggested that evolution of the C4 pathway was largely a story of gene duplication while plants were still in the ancestral C3 state [14].

Different genes in the C4 pathway were affected in different ways and at different times by gene duplication. Firstly, the approximately 70-mya pan-cereal WGD enriched the reservoir of some genes but not others. For example, in sorghum, both duplicated copies were preserved for PEPC and NADP-ME genes, and one of the copies of each gene produced by WGD was later recruited into the C4 pathway. This finding highlights the contribution of WGD to the evolution of C4 photosynthesis. However, for NADP-MDH, CA, PPDK-RP and PPCK enzyme genes, one of the WGD duplicates was probably lost. For CA, NADP-ME, and PPDK-RP, tandem gains of new genes after the sorghum-rice divergence appears to have preceded C4 evolution. This seems to suggest that earlier availability of the pan-cereal duplicated copies was not by itself sufficient to initiate C4 evolution, although it is not clear whether what was lacking was genetic (a part of the machinery) or environmental (a sufficiently strong selective advantage to drive the transition).

### Adaptive evolution of C4 genes

After duplication, there is evidence that some C4 genes experienced adaptive evolution; however, selection pressures and evolutionary modes have varied. Both maximum likelihood inference and patterns of aggregated amino acid differences indicate that the C4 NADP-ME and PPDK-RP enzyme genes have been under strong selective pressure. Maximum likelihood inference also implies that CA C4 enzymes have experienced positive selection, while aggregated amino acid differences indicate that C4 PEPC and PPDK genes may also have been under positive selection. The sorghum C4 genes of PPCK and NADP-MDH enzymes have also accumulated more substitutions than their rice orthologs, though the difference is not statistically significant. Compared to their rice orthologs, PEPC and NADP-MDH C4 genes evolve at a faster rate, providing further evidence of adaptation.

In many cases (NADP-ME, CA, PEPC) evidence from C4 plants supports adaptive evolution of the C4 gene family members only - the non-C4 homologs in C4 plants show no evidence of adaptive evolution, although the PEPC gene does show evidence in rice (C3). Further, the strongest evidence of adaptive evolution is in the period when the C4 pathway is thought to have evolved, after the divergence of sorghum and rice, but before the divergence of sorghum and maize.

Adaptive evolution is further supported by patterns of gene expression shown in previous reports. PEPC, PPDK, and CA C4 genes are expressed approximately 20 times more in sorghum mesophyll than bundle-sheath cells, while NADP-ME C4 genes are expressed much more in bundle-sheath than mesophyll cells [35]. The study of *Flaveria *intermediates shows that PEPC activity is increased approximately 40 times from C3 to full C4 species [65], and the NADP-ME activity is approximately 9 times higher in veins than mesophyll cells [66].

During the process of adaptive evolution, a duplicated gene may gradually acquire a new function (neofunctionalization) or subdivide the functions of its progenitor with the other duplicated copy (subfunctionalization). Laboratory evolution experiments indicated that an evolving new gene can initially acquire increased fitness for a new function without losing its original function [67]. This implies that a neofunctionalization process may begin with an initial subfunctionalization step, an implication that has been supported by theory [68]. It is unclear how long such a step may take. Here, we found that neofunctionalization of C3 genes to function in C4 photosynthesis could take a long time. Previous publications found that both C4 and non-C4 sorghum NADP-MDH genes were expressed in green leaves, though the C4 gene had higher transcript accumulation [43,44]. Together with maximum likelihood analysis involving more genes and different grasses, this finding indicated that C4 and non-C4 sorghum NADP-MDH genes, produced before sorghum-*Vetiveria *divergence, have experienced subfunctionalization [44]. Sequence alignment here indicates that the sorghum non-C4 gene has been affected by three insertions and one deletion in its amino-terminal coding sequence, suggesting functional innovation. Regardless of whether the process is a division of functions of their progenitor gene, or evolution of a novel function in the non-C4 gene, co-expression, albeit at divergent levels, of the two genes in green leaves suggests that the process may not yet be finished.

In addition to the possible sheltering effect of a duplicated copy when evolving genetic novelty, alternative splicing may further shelter functional changes. The maize PPDK gene (and probably also its sorghum ortholog) encoding C4 transcripts also encodes cytosolic transcripts. Their rice homolog also has a dual promoter [69], implying that natural selection may have utilized this pre-existing functional duality to evolve C4 function. If C4 transcripts are products of a novel function, and non-C4 transcripts due to the original function, the genes may have retained the original function for millions of years while evolving a novel function. The state of bifunctionality may continue until possible genetic incompatibility, if any, accumulates to a point intolerable to fitness. Maize PPDK may not be the only case of such gene bifunctionality in the C4 pathway. As shown above, the sorghum CA gene, Sb03g029170, seems to have similar bifunctionality, encoding both C4 and non-C4 transcripts. Since its internally repeating structure may have been produced before sorghum-maize divergence, its maize homologs also share this bifunctionality, which may have existed for millions of years. These multiple cases in which alternative splicing may contribute a possible sheltering effect during evolution of new function by C4 genes imply that it (alternative splicing) may participate in other cases of evolution of genetic novelty.

We found that the sorghum and maize C4 PEPC genes are on a long branch of their gene tree, grouped together with their suspected rice ortholog, showing possible adaptive evolution based on both a high Ka/Ks ratio and elevated amino acid substitution. It is intriguing to ask whether possible adaptive evolution in the rice PEPC gene could be a foundation toward a new origin of the C4 pathway, or instead indicates non-C4 functional adaptation. Scrutiny of the rice PEPC sequence revealed only 2 of 12 amino acid substitutions that were previously inferred to be positively selected in C4 genes [42], and, in particular, it lacks the critical fixed mutation S780 that is shared by C4 PEPC genes in other angiosperms [41,65]. This rice gene was classified into the *ppc-B1 *group [42], found only in the C3 grasses, suggesting that its adaptive evolution is not leading to C4 photosynthesis, but possibly to other functional novelty.

Adaptive evolution of PEPC may have some valuable implications for the discovery of multiple groups of PEPC genes defined previously [42]. In some C4 grasses there are different groups of genes, such as *ppc-B2 *and *ppc-C4*; while another group of genes, *ppc-B1*, are found in only C3 grasses. These findings show that, in the C4 lineages after their divergence from the C3 lineages but perhaps prior to the evolution of the C4 pathway itself, further gene duplication(s) may have contributed to the establishment of C4 photosynthesis.

### A novel mode of gene evolution

The CA enzyme genes display a novel mode of gene evolution and functional adaptation. As shown above, sorghum and maize C4 CA enzymes have one, two or three functional domains, produced through recursive duplications followed by a fusion process involving stop codon mutations in the leading domains. There have been at least four tandem duplication events in sorghum and its ancestral genomes. These tandem duplications started before sorghum-maize divergence, and appear to have continued in the maize lineage. The recurrence of tandem duplications together with the subsequent merger process may have acted as a mode of adaptive evolution. The present CA enzymes are beta-type, comprising a dimer having four zinc ions bound to the structure as active sites. Besides dimers, these enzymes can form tetramers, hexamers or octamers [47], suggesting that the dimer may be a building block. Recruiting extra domains through tandem duplication may contribute to the formation of more complex structures, with more functional binding sites making them work more efficiently to stabilize the balance between CO_2 _and . The expanded gene structure of these sorghum and maize CA genes are unusual, since the cDNAs of *Urochloa paniculata *and *Flaveria bidentis*, both C4 plants, are normal in size [70]. Nonetheless, there is precedent for internal repetition of CA gene structure in red algae, *Porphyridium purpureum*, resulting in two sets of functional binding sites [71]. This independent evolution of internally repeating structure in CA genes supports our hypothesis that such structure may confer functional advantages.

We found that the sorghum and maize C4 CA genes share a cTP, which had not been expected since the enzymes were not found to be chloroplastically localized in C4 plants. In C3 plants, the most abundant CA activity is in the chloroplast stroma, while in C4 plants, the exact location of CA is less clear [47], but the most abundant CA activity is localized in the cytosol of mesophyll cells [72]. The cTP of sorghum and maize C4 CA genes is similar to that of the *Arabidopsis *CA genes, suggesting its existence before monocot-dicot divergence. The preservation of a cTP in C4 genes for tens of millions of years cannot be explained as a mere relic but suggests possible multiple functionality. This inference is at least partially supported by the discovery of divergent functions implemented by two different transcripts produced by the single sorghum C4 CA gene, Sb03g029170. As shown above, the expression of the longer transcript is light-inducible, while that of the shorter one is not, indicating that the longer but not the shorter transcript may be involved in the C4 pathway.

### A long transition time from C3 to C4 photosynthesis

Several evolutionary models have been proposed to explain the formation of the C4 pathway [73-75]. In summary, seven significant phases are recognized toward successful establishment of C4 photosynthesis: general preconditioning (for example, gene duplication); anatomical preconditioning (for example, close veins); enhancement of bundle-sheath organelles; establishment of photorespiratory CO_2 _pump and transformation of glycine decarboxylase to bundle-sheath cells; enhancement of PEPC activity; integration; and optimization [6]. Although many biological and anatomical changes are needed, multiple origins in tens of angiosperm families suggest that it is not so difficult to evolve a novel C4 pathway. However, from an evolutionary viewpoint it is still interesting to ask whether a transition process of gene functional changes and/or enhancement is necessary before the final establishment, and how long such a transition might take. There has been a long time-lag between the initial decrease in CO_2 _concentration and the appearance of C4 plants. The initial decrease in CO_2 _concentration started at least 100 mya [6], while molecular clock analyses suggest that the earliest C4 plants (grasses) appeared about 24 to 35 mya [28,29] One proposed explanation for the time-lag was the lack of a sufficient reservoir of duplicated and neofunctionalized C3 genes to support C4 evolution [14]. Here, we found that the genes of key enzymes, such as PEPC and NADP-ME, were among the duplicated copies produced by the WGD approximately 70 mya [19,20]. Once again we note, however, that availability of the pan-cereal duplicated copies was not by itself sufficient to initiate C4 evolution, since some were lost from the common cereal ancestor and then, after divergence from rice, had to reduplicate in the sorghum-maize ancestor before C4 evolution could occur.

### Differential duplicability of C4 genes and their non-C4 isoforms

The above characterization of gene duplication shows differential duplicability of C4 genes and their isoforms in grasses. Evidence from yeast indicates that gene redundancy tends to be preserved among some of the central proteins in the cellular interaction network [76]. Tens of plant genes were suggested to be duplication-resistant, and undergo convergent restoration to singleton status following several independent genome duplications [25]. The differential duplicability could be explained by gene dosage effects, organismal complexity, protein interaction centrality and protein domain preference [24-26,76]. Here, we have shown that some gene families, including PEPC, PPCK, CA, and NADP-ME genes, have been expanded by gene duplication, but not others such as PPDK genes. The families expanded by gene duplication tend to be multiply functional, such as PEPC and NADP-ME [14]. Different PEPC gene isoforms take on specific roles, including the regulation of ion balance, the production of amino-group acceptor molecules in symbiotic nitrogen fixation, and the initial fixation of C in C4 photosynthesis and Crassulacean acid metabolism [77]. NADP-ME catalyzes the oxidative breakdown of malate to form CO_2 _and pyruvate in the C4 pathway. Its non-C4 functions include the provision of carbon skeletons for ammonia assimilation [78] and reductant for wound-induced production of lignin and flavonoids [79,80]. CA genes are also prone to duplication, which may enhance their ability to form more complex structures, as discussed above. Though further duplication is not required when a former C3 gene is finally co-opted for C4 roles [14], we found that the sorghum NADP-MDH C4 gene did experience a tandem duplication event, with only one duplicated copy preserving the C4 function through possible subfunctionalization [44]. This implies that the sorghum NADP-MDH C4 gene itself may be duplication-resistant.

### C4 pathway and codon usage bias

GC content elevation has resulted in codon usage bias [37], which has been hypothesized by some to have contributed to C4 adaptive evolution [30]. As shown above, though the grass C4 genes and their isoforms always have a higher GC content than their *Arabidopsis *counterparts, there is often a non-C4 grass gene having higher GC content than the C4 one(s). Thus, there is no clear evidence supporting co-variation between codon usage bias and C4 gene evolution. Base composition variation in grass genes has been a hot topic involving transcription, translation, modification and mutational bias [81-83].

### Potential contribution to engineering new C4 plants

A comprehensive characterization of the C4 pathway will help not only to understand how C4 photosynthesis evolves, but also may benefit crop improvement efforts. Of singular relevance are efforts to transform C3 plants into C4 plants. To perform such a transformation, one strategy is to incorporate the C4 pathway into C3 plants through recombinant DNA technology [84]. The strategy succeeds in transferring C4 genes into C3 plants and yielding high levels of C4 enzymes in desired locations [85,86]. It is of great interest to transform rice, a staple food for more than half of the world's population, to perform C4 function, as reviewed in a recent publication [31]. However, combined overproduction of C4 enzymes (PEPC, PPDK, NADP-ME, and NADP-MDH) resulted in only slightly higher levels of CO2 assimilation than in wild-type rice [87]. This might indicate that not all components needed for C4 photosynthesis are known. There must be some transporters involved and there might also be some unknown regulatory factors. Knowledge of the complete sorghum genome might help to identify such components. As also shown above, though often not statistically significant, the sorghum and maize C4 genes appear to have been under adaptive evolution in different modes and levels, and show different duplicability. These findings may provide clues toward a successful transformation to C4 photosynthesis. Alternatively, perhaps adaptations such as we have suggested in the PEPC gene in C3 lineages have mitigated the perceived weaknesses of C3 photosynthesis.

Subtle differences in the C4 pathways used in different grasses are worthy of further investigation as well. For example, if our hypothesis is correct that internally repeating structure in CA genes may confer functional advantages, then engineering of the maize trimer into sorghum (for example) may be advantageous. Likewise, exploration of still more recent polyploids such as sugarcane might yield even more complex CA alleles. Tandem duplication of C4 NADP-MDH following the sorghum-maize divergence does not appear to have been essential to C4 evolution; indeed, one of the tandem genes appears to have lost C4 specificity. However, careful scrutiny of the physiological consequences of this change might suggest benefits that could be transferred to other crops.

## Conclusions

### Gene duplication and C4 pathway evolution

Both WGD and single-gene duplication have contributed to C4 pathway evolution in sorghum and maize. Some C4 genes (PEPC, PPCK, and NADP-ME C4 genes) were recruited from duplicates produced by WGD. Sorghum NADP-MDH, NADP-ME and PPDK-RP C4 genes were affected by tandem duplication, with only one of the resulting copies involved in the C4 pathway. C4 genes show divergent duplicability. PEPC, NADP-ME, PPCK, and CA gene families were expanded by recursive duplication events, showing a duplication-philic nature, whereas NADP-MDH and PPDK are likely duplication-phobic. Further supporting evidence is that only one copy of NADP-MDH C4 gene duplicates preserves the C4 function.

### Adaptive evolution divergent in mode and level

We found evidence of adaptive evolution of most C4 genes studied. However, the mode and level of adaptation is divergent among C4 genes. Adaptive evolution is achieved though rapid mutations in DNA sequences, aggregated amino acid substitutions, and/or considerable increases of expression levels in specific cells. Besides gene redundancy, we found that alternative splicing may have also sheltered the evolution of new function. Our analysis supports previous findings that maximum likelihood inference may be too conservative to find adaptive evolution. We found no evidence of co-variation between codon usage bias and C4 pathway development.

### Special evolutionary mode of grass CA genes

Grass CA genes have evolved in a specific pattern featuring recursive tandem duplication and neighboring gene fusion, which produced distinct isoforms having one to three functional units. Two sorghum C4 CA genes have one and two functional units, while two characterized maize C4 CA genes have two and three functional units, respectively. The elongation of these genes by recruiting extra domains may contribute to the formation of more complex protein structures, as often observed in plants.

### A long transition time from C3 to C4 photosynthesis

The hypothesis that a reservoir of duplicated genes in ancestral C3 plants was a prerequisite for C4 pathway development is only partially supported by present findings that some C4 genes were recruited from the duplicates. Availability of the pan-cereal duplicated copies was not sufficient to initiate C4 evolution, since some were lost from the common cereal ancestor, then had to reduplicate in the sorghum-maize ancestor before C4 evolution could occur. However, C4 gene isoforms show quite divergent duplicability, and there has been quite a long time-lag between the gene duplication events and the appearance of C4 grasses. These findings suggest a long transition process, including different modes of functional innovation, before the eventual establishment of C4 photosynthesis.

## Materials and methods

Known C4 enzyme genes and their non-C4 isoforms in sorghum, maize and *Arabidopsis *(Table 1) were downloaded from NCBI CoreNucleotide database [88]. Searching these known genes against sorghum [89] and rice [90] gene models by running BLAST [91] (E-value < 1 × 10^-5^), we identified other enzyme genes in these organisms. By characterizing sequence similarity and constructing gene trees, possible C4 genes were determined. The enzymes revealed here were linked to expression data reported previously [35] by comparing cDNA segments to gene sequences using BLAST.

### Gene colinearity inference

The potential gene homology information defined by running BLAST was used as the input for MCscan [92] to find homologous gene pairs in colinearity. The built-in scoring scheme for MCscan is min(-log_10_*E_value*, 50) for every matching gene pair and -1 for each 10 kb distance between anchors, and blocks that had scores >300 were kept. The resulting syntenic chains were evaluated using a procedure by ColinearScan [17] and an E-value < 1 × 10^-10 ^was used as a significance cutoff.

### Gene phylogeny construction

We constructed phylogenetic trees using several approaches, including the neighbor-joining, maximum likelihood, minimal evolution, and maximum parsimony methods, implemented in NADP-MEGA [93], PHYML [94], and PHYLIP [95], on both DNA and protein sequences. While running PHYML, parameters were set as adopted previously [42]. Bootstrap tests were performed with 100 repeats to produce percentage values, showing the stability of their topology. The trees mostly agree with one another. When there is inconsistency, the tree most strongly supported by bootstrap values was adopted for the subsequent adaptive evolution inference. For example, the trees of CA functional units were inconsistent among methods, and the best-supported neighbor-joining tree produced by protein sequences was adopted for further analysis.

### Maximum likelihood inference of adaptive evolution

The tree constructed for the group of C4 enzyme genes and their non-C4 isoforms was used to perform further maximum likelihood analysis using the Codeml program in PAML [96]. To detect whether a specific C4 gene had been positively selected, we compared two types of competing models: a free-ratio model and a ratio-restriction model [97]. The free-ratio model assumes an independent Ka/Ks ratio for each branch, whereas the latter forces the Ka/Ks ratio to be 1 on the specific branch to be tested for positive selection, and for the other branches assumes independent ratios. Each model will produce a likelihood, and the twofold difference between them follows a Chi-squared distribution with 1 degree of freedom.

### Aggregated amino acid substitution analysis

We adopted a comparative genomic approach initially proposed by Wagner [98] to detect genes potentially under positive selection. The Wagner approach inferred positive selection pressure by detecting possible aggregation of amino acid replacement. Here, we inferred possible amino acid replacements by comparing the homologous enzyme gene pair containing a C4 gene and non-C4 gene (often a rice gene) against the aligned outgroup sequence. A replacement site is identified in the C4 sequence that differs from the corresponding sites in both the homologous sequence and the outgroup sequence, which are identical. We found the number of all replacements, m, grouping the C4 and non-C4 protein sequences. If the occurrences of these replacement sites are assumed to be Poisson distributed with a parameter *λ*, we may evaluate the chance of observing a specific number of consecutive replacement sites along a sequence. For simplicity in description, for each sequence we first defined a replacement position array, *x *= *x*_0_, *x*_1_, *x*_2_,..., *x*_*t*_, *x*_*t*+1_, composed by all the positions *x*_*i *_(1 ≤ i ≤ *t*) of replacement sites and two ends of the sequence, that is, *x*_0 _= 0 and *x*_*t*+1 _= *n *- 1, where *n *is the length of the alignment after purging gaps. Then we defined the replacement distance array, *d *= (*d*_1_,... *d*_*t*+1_), where *d*_1 _= *x*_*i *_- *x*_*i*-1 _(1 ≤ i ≤ *t *+ 1). The distance between two replacement sites *d*_*i*, *k *_= *x*_*i*+*k*-1 _- *x*_*i*_, where *k *is the number of the consecutive replacement sites in the corresponding sequence segment, follows a Pearson type III distribution following a probability density *λ*(*λz*)^*k*-2 ^*e*^-*λz*^/Γ(*k *- 1) [99], where Γ(*k *- 1) = (*k *- 1)!. We can estimate the Poisson parameter *λ *with *m*/(*2n*). Supposing there are *t*_*i *_replacement sites along the *i*-th sequence, obviously, we get . Therefore, we could estimate the probability *P*(*d*_*i*, *k*_) that *k *consecutive replacements in a distance between two replacement sites is smaller than the observed *d*_*i*, *k *_by the following integration:



We evaluated the occurrence probability of observed distance between any two replacement sites, and the smallest probability was used to locate a region with the most aggregated replacements, which was taken to be significant after a Bonferroni correction by considering the number of all combinations of replacement sites (). The occurrence probability was calculated using R [100]. If between two replacement sites there were gaps in the aligned sequences, they were omitted to check for possible selection. We composed Perl scripts to implement the described approach.

### Maize homolog characterization

Maize BACs are from the MaizeSequence database [101]. The maize genes were searched against the BAC sequences to reveal their chromosomal locations, local DNA structures, and so on.

### Chloroplastic transit peptide inferrence

ChloroP1.1 [102] was used to predict the presence of cTPs in the enzyme protein sequences and the location of potential cTP cleavage sites

### Dotplotting

Dotplots between CA protein sequences were produced by running the public program DOTTER [103]. The Dotplots were produced by matched strings from two protein sequences in comparison. The expected score per residue of the matched strings was set to be 40.

## Abbreviations

BAC: bacterial artificial chromosome; CA: carboxylating anhydrase; cTP: chloroplast transit peptide; MDH: malate dehydrogenase; mya: million years ago; NADP-ME: NADP-malic enzyme; PEPC: phosphoenolpyruvate carboxylase; PPCK: PEPC kinase; PPDK: pyruvate orthophosphate dikinase; PPDK-RP: PPDK regulatory protein; WGD: whole-genome duplication.

## Authors' contributions

XW designed and organized the present work. UG, PW and XW curated gene models. UG, HT, JEB and AHP contributed to this work through critical discussion. XW and AHP wrote the paper.

## Additional files

The following additional data are available with the online version of this paper: Tables S1 to S4 (Additional data file 1); a full tree of PEPC genes (Additional data file 2); cTPs detected by ChloroP (Additional data file 3); CA gene sequences and their functional units (Additional data file 4).

## Supplementary Material

Additional File 1 Table S1: C4 gene isoforms in the present study. Table S2: sorghum C4 genes with cDNA evidence; Table S3: GC content of C4 gene isoforms. Table S4: complete results of inferred amino acid substitutions and their aggregation. Click here for file

Additional File 2 Full tree of PEPC genes. Click here for file

Additional File 3 cTPs detected by ChloroP. Click here for file

Additional File 4 CA gene sequences and their functional units. Click here for file
