# Supplementary material for: Comparative genomic analysis of C4 photosynthetic pathway evolution in grasses
Source: Genome Biol. 2009 Jun 23;10(6):R68. doi: 10.1186/gb-2009-10-6-r68 (PMC2718502; doi:10.1186/gb-2009-10-6-r68)
Supplement: Additional File 2 — Full tree of PEPC genes. [file gb-2009-10-6-r68-S2.doc]

Additional figure 1. Phylogenetic trees containing sorghum, rice, maize and *Arabidopsis* genes encoding PEPC. The trees were constructed with neighbor-joining (NJ) and maximal likelihood (PHYML) approaches. (A) NJ approach on protein sequences; (B) PHYML approach (JTT+I+G model) on protein sequences; (C) PHYML approach (GTR+I+G model) on nucleotide coding sequences; After removing a subgroup of three genes possibly produced by duplication occurred before monocot-dicot divergence: (D) NJ approach on protein sequences; (E) PHYML approach (GTR+I+G model) on nucleotide sequences. Parameters when running PHYML can be found at website: http://atgc.lirmm.fr/phyml/.

(A).

(B).

(C).

(D).

(E).
